# Factors associated with fatigue in patients with systemic lupus erythematosus in an outpatient tertiary care setting: a cross-sectional cohort study

**DOI:** 10.1136/bmjopen-2025-104786

**Published:** 2026-04-01

**Authors:** Marquis Chapman, Ali M Rizvi, Miraa Qutab, Adam Munday, Ann Biehl, Zerai Manna, Sarfaraz Hasni

**Affiliations:** 1 Lupus Clinical Trials Unit, Systemic Autoimmunity Branch, National Institute of Arthritis and Musculoskeletal and Skin Diseases, National Institutes of Health, Bethesda, Maryland, USA; 2 Post Graduate Medical Institute, Lahore, Punjab, Pakistan; 3 Clinical Center Pharmacy Department, National Institutes of Health Clinical Center, Bethesda, Maryland, USA

**Keywords:** Fatigue, Patient Reported Outcome Measures, RHEUMATOLOGY

## Abstract

**Abstract:**

**Objectives:**

Fatigue is one of the most common and debilitating symptoms experienced by patients with systemic lupus erythematosus (SLE). Previous studies revealed the association of fatigue with various SLE and non-SLE-related factors. This study aims to explore the prevalence of fatigue and the factors that are associated with fatigue experienced by SLE patients in an outpatient rheumatology clinic setting.

**Design:**

Prospective, observational study using a sample of convenience.

**Setting:**

Outpatient rheumatology clinic at a tertiary care centre.

**Participants:**

Consecutive subjects with SLE presenting for their outpatient visits enrolled in the ongoing Institutional Review Board-approved ‘Pathogenesis and Natural History of SLE’ protocol.

**Primary and secondary outcome measures:**

Disease activity and organ damage accrual were measured by Safety of Estrogens in Lupus Erythematosus: National Assessment Version of the Systemic Lupus Erythematosus Disease Activity Index (SELENA-SLEDAI) and Systemic Lupus International Collaborating Clinics/American College of Rheumatology Damage Index (SLICC/ACR DI), respectively. Fatigue was measured by the self-reported Fatigue Severity Scale (FSS), and a score of ≥4 was used to define clinically significant fatigue. Correlation analyses were done to determine the association between fatigue and patient demographics, and SLE disease activity and damage indices. Results were considered as statistically significant at p<0.05. All data analyses were carried out with the SAS program, release V.9.4 (SAS Institute).

**Results:**

183 patients completed the study, with a significant proportion (144/183) belonging to ethnic minorities. The overall FSS score was mean (±SD) 4±1.8 and SELENA-SLEDAI score of 3±2.6. The group reporting significant FSS scores ≥4 (N=95) included a higher proportion of White patients, more organ damage (SLICC/ACR DI score mean (±SD) 1.9±1.9) and higher body mass index (BMI) mean (±SD) 29.6±6.7 kg/m^2^; as compared with the group with FSS scores <4(N=85), which had a higher proportion of Black patients (p=0.034), lower SLICC/ACR DI scores (1.1±1.3 (p=0.008)) and BMI 27.6±5.6 kg/m^2^ (p=0.03).

**Conclusions:**

Our study found that organ damage accrual, specifically pulmonary fibrosis and neuropathy as measured by SLICC-ACR DI and high BMI, is associated with clinically significant fatigue in SLE. Furthermore, our results support previous findings that fatigue is independent of SLE disease activity. Findings of our study need to be replicated in independent SLE cohorts measuring fatigue at multiple time points. Mechanistic studies are needed to better understand pathogenesis of fatigue in SLE.

**Strengths and limitations of this study**

- The cohort of patients was significantly more diverse than prior studies on fatigue in systemic lupus erythematosus (SLE).
- Most of the subjects had SLE for a long duration.
- Most subjects had minimal SLE disease activity at the time of study participation.
- Limitations of our methods include being a single-centre, cross-sectional study.

## Introduction

 Fatigue is omnipresent and debilitating in patients with systemic lupus erythematosus (SLE), with more than 80% of patients reporting some degree of fatigue and over half indicating fatigue as their most disabling symptom.^1 2^ Fatigue in SLE has been defined as a feeling of whole-body tiredness that is disproportionate to the energy expenditure.^3^ Fatigue limits patients’ ability to perform activities of daily living and consequently reduces overall quality of life and has an additional socioeconomic impact. Health-related quality of life (HRQoL) at least partially as a result of fatigue is also substantially reduced in those with SLE, and to a degree that is even more dramatic than that observed in Rheumatoid Arthritis.^4^,^9^ Fatigue has been identified as the most difficult symptom to live with by 91% of surveyed individuals with SLE in the UK.^10^ Additionally, it has been reported that lower levels of physical and cognitive functioning in SLE patients reduce employment, with studies from the USA, Canada and Sweden reporting unemployment rates for the SLE population up to more than 40%.^11^,^15^ High unemployment rates may partially be due to disease damage accrual and activity; however, fatigue impacts work performance and has been reported as the primary determinant of unemployment and low work productivity.^16 17^

Pathophysiological mechanisms underlying fatigue in SLE are not well defined. Dysregulation of mitochondrial structure and function leads to increased oxidative stress, which contributes to inflammation and fatigue seen in SLE.^18^ An altered kynurenine pathway, leading to an increased kynurenine/tryptophan ratio, was shown to correlate with fatigue in SLE.^19^ High levels of quinolinic acid, a metabolite in the kynurenine pathway, were reported in patients with SLE. Quinolinic acid has been shown to initiate inflammation and can generate reactive oxygen species, resulting in fatigue. Quinolinic acid is required for the synthesis of nicotinamide adenine dinucleotide (NAD+), a coenzyme that catalyses redox reactions in the body.^20^ Boosting NAD+blunts attenuated type I IFN signalling in myeloid cells and may improve mitochondrial dysfunction and fatigue in SLE patients.^21^ A clinical trial to evaluate the effect of NAD+boosting in SLE is currently underway (*NCT06032923*). Multiple factors, such as physical activity, pain, fibromyalgia, depression, body mass index (BMI), age, education level and health insurance status, have been associated with fatigue in SLE in previous studies.^22^,^24^ Data reported from the Toronto Lupus Cohort and the Lupus in Minorities: Nature vs nurture (LUMINA—a multiethnic US cohort) suggest that lupus disease activity or damage accrual is not associated with fatigue in SLE.^22^ Conversely, the results from clinical trials for belimumab showed a significant improvement in fatigue in patients who had an improvement in SLE Responder Index (SRI-4), suggesting a positive correlation between fatigue and lupus disease activity.^25^ There are also reports that associate fatigue severity with the damage accrual as measured by the Systemic Lupus International Collaborating Clinics/American College of Rheumatology Damage Index (SLICC/ACR DI).^26 27^

The aim of the current study is to explore the prevalence of fatigue and the variables associated with fatigue in a cohort of lupus patients presenting to a tertiary care outpatient rheumatology clinic. We looked at the association of fatigue with lupus disease duration, activity, damage, medications and non-lupus-specific factors such as demographic factors, BMI and blood haemoglobin levels.

## Methods

### Subjects

All subjects, after providing informed consent, were consecutively recruited from an outpatient clinic at a tertiary research facility over a period of 18 months. All subjects met the 1997 ACR Revised Criteria for the Classification of SLE.^28 29^ The subjects were approached to fill out the Fatigue Severity Scale (FSS) at the time of their routine outpatient follow-up visit. An interpreter was used for non-English-speaking subjects when a validated version of the FSS was not available in the preferred language. No subject was excluded from the study based on their language preference.

### Study design

A convenience sampling method was used for this exploratory study. All data were collected and recorded during the routine outpatient visits and included demographic information, FSS composite scores, BMI calculations, and SLE clinical and serological disease activity and damage accrual by calculating Safety of Estrogens in Lupus Erythematosus: National Assessment Version of the Systemic Lupus Erythematosus Disease Activity Index (SELENA-SLEDAI) and SLICC/ACR DI scores. The rheumatologist responsible for clinical assessment and determining SELENA-SLEDAI and SLICC/ACR DI was blinded to the FSS scores. Comorbidities and use of concomitant medications were also collected on the day of the visit. Current prednisone use and dose were recorded on the day of the visit. Lab parameters, including haemoglobin level and complement proteins, with potential effects on fatigue, were obtained on the day of the visit.

#### Fatigue Severity Scale

The FSS is a well-documented and reliable tool for measuring fatigue in patients with SLE^29 30^ and the most commonly used scale for assessing patient-reported fatigue in research protocols recruiting individuals who have SLE.^30^ The FSS is a nine-item tool with a minimum score of one and a maximum score of seven. Perceived fatigue increases with increases in numerical score. Scores across the nine items are averaged to create a composite score of between one and seven. A composite FSS score ≥4 is considered to be clinically significant fatigue.^31^

#### SELENA-SLEDAI and SLICC-ACR damage indices

These indices are determined by rheumatologists who previously underwent training and demonstrated competency in score calculation. A SLEDAI score of four or more was considered to reflect active disease.^32^ The SLICC-ACR DI is a measure of absolute damage accrual that occurs in SLE patients. It is generally a non-decreasing index, which reflects the accumulation of non-reversible damage and the disease progression. In order for an item to be considered, it must be present for 6 months or more unless it is specified otherwise in the index. The SLICC-ACR DI covers 12 organ systems in the body. It is important to note that the source of the damage, either directly from lupus or otherwise, does not affect whether it is added to the damage index of the patient.

### Statistical analysis

Subject demographics were characterised and reported by mean, SD and range for the continuous variables, and frequencies and percentages for the categorical variables. Independent samples t-tests were performed to compare continuous variables between the high and low FSS score groups, while chi-square analyses were conducted to assess differences in categorical variables between the groups. Logistic regression analysis was performed to identify independent predictors of high versus low FSS scores. A multiple regression model was fitted, paying special attention to multicollinearity. Spearman’s rank correlation coefficient was used to examine the strength and direction of associations between fatigue and individual organ system scores of the SLICC/ACR Damage Index, as the data were not normally distributed. Stepwise multivariable regression analyses were performed to identify factors associated with fatigue in SLE patients. Individual items from the SLICC/ACR Damage Index were included as candidate predictors, and models were adjusted for key confounders, including age and disease duration, due to their known associations with accumulated organ damage. The independent factors are the continuous variables, including SELENA-SLEDAI, SLICC ACR DI, disease duration, BMI and haemoglobin level. Results were considered as statistically significant at p<0.05. All data analyses were carried out with the SAS program, release V.9.4 (SAS Institute).

### Patient and public involvement

Patients and the public were not involved in study design, conduct, dissemination of results or evaluation of studies.

## Results

### Patient characteristics

A total of 183 subjects, mostly women (N=174), completed the study (table 1).

**Table 1:** Demographic characteristics of the participants (N=183)

Age (years) Mean±SD	45.6±12.7
Disease duration (years) Mean±SD	13.8±10.5
Race, N (%)	
Black	44 (24.0%)
Asian	21 (11.5%)
White	39 (21.3%)
Hispanic	79 (43.2%)
Sex, N (%)	
Female	174 (95.1%)
Male	9 (4.9%)
FSS Score Mean±SD	4.0±1.8
SELENA-SLEDAI Score Mean±SD	3±2.6
SLICC/ACR DI Mean±SD	1.6±1.7
BMI kg/m^2^ Mean±SD	28.7±6.2
Prednisone (mg/day) Mean±SD	5.6±6.1
Haemoglobin (g/L), Mean±SD	123±15
HCQ N (%)	
Yes	154 (84.1%)
No	29 (15.9%)
Prednisone (Y/N) N (%)	
Yes	131 (71.6%)
No	52 (28.4%)
AZA N (%)	
Yes	35 (19.1%)
No	148 (80.9%)
MTX N (%)	
Yes	18 (9.8%)
No	165 (90.2%)
MMF N (%)	
Yes	47 (25.7%)
No	136 (74.3%)
Depression N (%)	
Yes	16 (8.7%)
No	167 (91.3%)
Hypothyroidism N (%)	
Yes	24 (13.1%)
No	159 (86.9%)
Cardiovascular disease N (%)	
Yes	9 (4.9%)
No	174 (95.1%)
Respiratory disease N (%)	
Yes	16 (8.7%)
No	167 (91.3%)
Complement*	
Low	66 (36.1%)
Normal	117 (63.9%)
Anti-ds DNA†	
Positive	81 (44.3%)
Negative	102 (55.7%)

* Complement levels at the time of FSS survey.

† Anti-ds-DNA levels at the time of FSS survey.

AZA, azathioprine; BMI, body mass index; FSS, Fatigue Severity Scale; HCQ, hydroxychloroquine; MMF, mycophenolate mofetil; MTX, methotrexate; SELENA-SLEDAI, Safety of Estrogens in Systemic Lupus Erythematosus National Assessment-Systemic Lupus Erythematosus Disease Activity Index; SLICC ACR DI, Systemic Lupus International Collaborating Clinics/American College of Rheumatology Damage Index.

The mean (±S) age was 45.6±12.7 years with a mean (±SD) disease duration of 13.8±10.5 years. The majority of subjects (79.7%) were predominantly Hispanic (43.2%) or African American (AA) (24%). Most of the lab parameters were in the normal range (online supplemental table 1). The overall cohort reported clinically significant fatigue with a composite FSS score of 4.0±1.8 (mean (±SD)) and low disease activity SELENA-SLEDAI Score 3±2.6 (mean±SD). There were 81 (44.2%) patients in remission due to SLEDAI ≤4 with no clinical disease activity, and Physician’s Global Assessment (PGA) of <0.5. The majority of SLE clinical activity was due to rash, alopecia and renal involvement. The clinical domains for patients with SELENA-SLEDAI >4 are listed in online supplemental table 2.

The subjects were stratified into low and high fatigue by using a cut-off of an FSS score of ≥4. The low FSS group (N=88) and high FSS group (N=95) were similar in age, disease, duration and disease activity (table 2).

**Table 2:** Comparison of subjects with low versus high fatigue

	FSS Low (FSS <4)	FSS High (FSS ≥4)	All subjects	P value
Age (years)	(N=88)	(N=95)	(N=183)	0.22
Mean (SD)	44.4 (13.1)	46.7 (12.2)	45.6 (12.7)	
Disease duration (years)				
Mean (SD)	13 (9.3)	14.6 (11.4)	13.8 (10.5)	0.3
Race/ethnicity, N (%)				
Black	26 (14.21%)	18 (9.84%)	44 (24.04%)	0.034
Asian	11 (6.01%)	10 (5.46%)	21 (11.48%)	
White	11 (6.01%)	28 (15.3%)	39 (21.31%)	
Hispanic	40 (21.86%)	39 (21.31%)	79 (43.17%)	
Sex, N (%)				0.07
Female	93 (50.8%)	81 (44.3%)	174 (95.1%)	
Male	2 (1.1%)	7 (3.8%)	9 (4.9%)	
SELENA-SLEDAI Score				0.89
Mean (SD)	3 (2.6)	3 (2.7)	3 (2.6)	
SLICC ACR DI score				
Mean (SD)	1.1 (1.3)	1.9 (1.9)	1.6 (1.7)	0.0008
BMI kg/m^2^				
Mean (SD)	27.6 (5.6)	29.6 (6.7)	28.7 (6.2)	0.03
Prednisone (mg/day)				
Mean (SD)	4.9±4.8	6.2±7.1	5.6±6.1	0.16
Haemoglobin (g/L)				
Mean (SD)	123 (16)	123 (14)	123 (15)	0.85
HCQ				
Yes	74 (40.4%)	80 (43.7%)	154 (84.1%)	1.00
No	14 (7.6%)	15 (8.2%)	29 (15.9%)	
Prednisone (Y/N)				
Yes	63 (34.4%)	68 (37.2%)	131 (71.6%)	1.00
No	25 (13.7%)	27 (14.8%)	52 (28.4%)	
AZA				0.19
Yes	13 (7.1%)	22 (12.02%)	35 (19.1%)	
No	75 (40.98%)	73 (39.89%)	148 (80.9%)	
MTX				0.62
Yes	10 (5.5%)	8 (4.4%)	18 (9.8%)	
No	78 (42.6%)	87 (47.5%)	165 (90.2%)	
MMF				0.06
Yes	17 (9.3%)	30 (16.4%)	47 (25.7%)	
No	71 (38.8%)	65 (35.5%)	136 (74.3%)	
Depression				0.2
Yes	5 (2.7%)	11 (6.0%)	16 (8.7%)	
No	83 (45.4%)	84 (45.9%)	167 (91.3%)	
Hypothyroidism				0.28
Yes	9 (4.9%)	15 (8.2%)	24 (13.1%)	
No	79 (43.2%)	80 (43.7%)	159 (86.9%)	
Cardiovascular disease				0.50
Yes	3 (1.6%)	6 (3.3%)	9 (4.9%)	
No	85 (46.5%)	89 (48.6%)	174 (95.1%)	
Respiratory disease				0.44
Yes	6 (3.28%)	10 (5.46%)	16 (8.74%)	
No	82 (44.81%)	85 (46.45%)	167 (91.26%)	
Complement				0.539
Low	34 (18.6%)	32 (17.5%)	66 (36.1%)	
Normal	54 (29.5%)	63 (34.4%)	117 (63.93%)	
Anti-ds DNA				0.555
Positive	41 (22.4%)	40 (21.9%)	81 (44.3%)	
Negative	47 (25.7%)	55 (30.1%)	102 (55.8%)	

FSS high and low score groups were compared age, SLEDAI, SLICC ACR DI, BMI and disease duration, using independent sample t-tests for the continuous data. χ2 analyses are used on the level of measurement for the categorical data, such as ethnicity and sex.

Lab values measured at the time of FSS survey.

P value refers to the comparison between the levels of fatigue.

AZA, azathioprine; BMI, body mass index; FSS, Fatigue Severity Scale; HCQ, hydroxychloroquine; MMF, mycophenolate mofetil; MTX, methotrexate; SELENA-SLEDAI, Safety of Estrogens in Systemic Lupus Erythematosus National Assessment-Systemic Lupus Erythematosus Disease Activity Index; SLICC ACR DI, Systemic Lupus International Collaborating Clinics/American College of Rheumatology Damage index.

The proportion of non-Hispanic White subjects reporting clinically significant fatigue (FSS≥4) was higher as compared with the proportion of black subjects who reported FSS≥4 . The group with higher FSS scores had accrued more damage, with a mean SLICC/ACR DI score of 1.9±1.9 (SD), and had a higher mean BMI (29.6±6.7); in comparison, the group reporting less fatigue (FSS score <4) had less damage accrual, with a mean SLICC/ACR DI score of 1.1±1.3 SD (p=0.0008) and had a lower mean BMI of 27.6±5.6 (SD), (p=0.03). The majority of patients did not have any comorbid conditions that could contribute to fatigue. In the subset of patients with depression, hypothyroidism, cardiovascular or respiratory diseases, there were more who reported clinically significant fatigue, but the results were not statistically significant (table 2). The information about the socioeconomic background was not collected for this study. The current use and dose of prednisone were similar in both groups. Comparison of various lab parameters did not reveal any significant difference between the two groups (online supplemental table 3).

### Variables associated with clinically significant fatigue

The logistic regression models were used to determine which variables were associated with high versus low FSS scores (table 3).

**Table 3:** Factors associated with FSS using logistic regression model

Factors	OR	Coefficient (95% Cl)		Significance
		Lower	Upper	(P value)
Marital status	0.853	0.648	1.124	0.26
Ethnicity Black vs non-Black	1.818	0.776	4.26	0.17
Ethnicity White vs non-White	0.338	0.133	0.859	0.02
Age	0.996	0.965	1.029	0.83
SLICC ACR DI Score	1.458	1.135	1.873	0.003
SELENA-SLEDAI SCORE	0.976	0.838	1.137	0.76
BMI	1.072	1.011	1.138	0.02
Disease duration	0.978	0.94	1.019	0.29
Haemoglobin	0.992	0.786	1.253	0.95
HCQ	0.842	0.339	2.091	0.71
Prednisone	1.286	0.571	2.899	0.54
C3	0.986	0.967	1.006	0.16
C4	1.029	0.981	1.08	0.24

Using the cut-point score of 4 on FSS used low n=88 and high n=95 groups, logistic regression models were used to determine whether marital status, ethnicity (Black vs non-Black), age, SLICC/ACR DI, SELENA-SLEDAI, BMI, duration of disease, haemoglobin, hydroxychloroquine, prednisone, C3 and C4 were independent predictors of binary dependent variable FSS high and low scores. Variable were not highly correlated, indicating no threat of multicollinearity in the model.

BMI, body mass index; C4, complement component 4; FSS, Fatigue Severity Scale; HCQ, hydroxychloroquine; SELENA-SLEDAI, Safety of Estrogens in Systemic Lupus Erythematosus National Assessment-Systemic Lupus Erythematosus Disease Activity Index; SLICC ACR DI, Systemic Lupus International Collaborating Clinics/American College of Rheumatology Damage Index.

When all ethnicity variables were assessed simultaneously in logistic regression analysis, none of them reached statistical significance. On comparing each ethnicity individually, the White versus non-White and Black versus non-Black variables achieved statistical significance (p=0.006* and p=0.03*, respectively). Based on these findings, both the White vs Non-White and Black versus non-Black variables were retained in the final model.

In a multivariate model, damage accrual SLICC/ACR DI(OR=1.42, 95% CI 1.11 to 1.81, p=0.004), BMI (OR=1.06, 95% CI 1.01 to 1.13, p=0.02) and White race (OR=0.34, 95% CI 0.133 to 0.85, p=0.02) were found to have a statistically significant association with clinically significant fatigue, after adjusting for confounders (table 4).

**Table 4:** Multivariate regression model results predicting fatigue adjusted for confounders

Variable	β, beta estimate	SE	95% CI		P value
			Lower	Upper	
SELENA- SLEDAI	0.02349	0.04987	−0.075	0.122	0.6381
SLICC ACR DI	0.29042	0.08645	0.120	0.461	0.0010
Disease duration (years)	−0.01072	0.01392	−0.038	0.017	0.4424
BMI, kg/m^2^	0.04524	0.02044	0.005	0.086	0.0282
Hgb, g/L	0.00918	0.08476	−0.158	0.176	0.9139

BMI, body mass index; Hgb, haemoglobin; SELENA-SLEDAI, Safety of Estrogens in Systemic Lupus Erythematosus National Assessment-Systemic Lupus Erythematosus Disease Activity Index; SLICC ACR DI, Systemic Lupus International Collaborating Clinics/American College of Rheumatology Damage Index.

The SELENA-SLEDAI score, disease duration, serum haemoglobin, use of prednisone, hydroxychloroquine or serum complement proteins C3 and C4 were not found to be associated with clinically significant fatigue.

### SLICC/ACR DI items associated with fatigue

There were 64 patients with no damage accrual based on the SLICC ACR DI score of 0, whereas 119 patients had a score of ≥1. We did a correlation analysis to identify the relationship between individual SLICC ACR DI items and FSS (table 5).

**Table 5:** Correlation between fatigue and individual SLICC ACR DI items

SLICC ACR DI items	r	P value	Number of individuals where item was scored
Pulmonary fibrosis	0.19423	0.008	11
Vascular tissue loss	0.16389	0.027	4
Pulmonary hypertension	0.15737	0.033	9
Neuropathy cranial	0.15091	0.041	5
Diabetes	0.14893	0.044	11
Osteoporosis	0.14643	0.048	7
Malignancy	0.14466	0.051	11
Cerebrovascular accident	0.13607	0.066	8
Pulmonary shrink lung	0.13394	0.071	3
Cardiomyopathy	0.12855	0.083	3
Gonadal failure	0.1172	0.114	8
Glomerular rate	−0.1171	0.114	18
Avascular necrosis	0.10689	0.150	14
Cognitive impairment	0.09649	0.194	9
Myocardial infarction	0.0957	0.198	3
Pericarditis	−0.0842	0.257	3
Renal disease end	−0.08126	0.274	3
Arthritis deform	0.07879	0.289	13
Oesophageal stricture	0.07536	0.311	1
Proteinuria	0.06016	0.419	2
Alopecia	0.04802	0.519	13
Seizure therapy	0.04626	0.534	8
Venous thrombosis	0.04518	0.544	14
Transverse myelitis	0.04299	0.563	4
Muscle atrophy	0.03931	0.597	6
Cardio angina	0.031	0.677	9
Pulmonary infarction	−0.01837	0.805	1
Pulmonary pleural fibrosis	0.01414	0.849	1
Retinal change	−0.0096	0.897	22
Osteomyelitis	0.00949	0.899	1
Cataract	0.0049	0.948	31
Skin extensive scar	−0.00374	0.960	5

Pearson correlation coefficient was used to describe the linear relationship between FSS versus the SLICC ACR/DI items.

FSS, Fatigue Severity Scale; SLICC ACR DI, Systemic Lupus International Collaborating Clinics/American College of Rheumatology Damage Index.

Multiple items were found to have significant correlation with FSS, such as pulmonary fibrosis (p=0.008), pulmonary hypertension (p=0.033), vascular tissue loss (p=0.027) and neuropathy (p=0.041). In addition, diabetes (p=0.044) and osteoporosis (p=0.048) were also significantly correlated with fatigue. By using a stepwise multivariable regression model, the only variables that remained significantly associated with FSS were neuropathy (p=0.017, 95% CI 0.42 to 4.19) and pulmonary fibrosis (p=0.034, 95% CI 0.11 to 2.92) (table 6).

**Table 6:** Stepwise multivariable regression model to identify individual SLICC ACR DI items predictive of fatigue

SLICC ACR DI items	β, beta estimate	SE	95% CI		P value
			Lower	Upper	
Neuropathy	2.31	0.95	0.42	4.19	0.017
Pulmonary fibrosis	1.40	0.65	0.11	2.69	0.034
Pulmonary shrinking lung	1.98	1.05	−0.10	4.07	0.061
Glomerular filtration rate	−0.68	0.37	−1.41	0.06	0.070
Cardiomyopathy	1.94	1.18	−0.39	4.28	0.10
Gonadal failure	1.06	0.68	−0.28	2.40	0.12
Pulmonary pleural fibrosis	−3.03	2.03	−7.05	0.99	0.14
Cataract	−0.61	0.41	−1.42	0.21	0.14
Oesophageal stricture	2.68	1.87	−1.02	6.39	0.15
Renal disease end	−0.46	0.35	−1.15	0.24	0.19
Diabetes	0.74	0.58	−0.41	1.89	0.20
Malignancy	0.64	0.50	−0.35	1.62	0.20
Proteinuria	1.49	1.27	−1.01	3.99	0.24
Vascular tissue loss	1.06	1.01	−0.94	3.05	0.30
Cerebrovascular accident	0.52	0.53	−0.52	1.56	0.33
Alopecia	0.50	0.54	−0.56	1.56	0.35
Pericarditis	−0.86	1.03	−2.89	1.17	0.40
Pulmonary hypertension	0.62	0.74	−0.84	2.07	0.40
Osteoporosis	0.70	0.85	−0.97	2.38	0.41
Transverse myelitis	0.81	0.99	−1.15	2.77	0.42
Avascular necrosis	0.26	0.34	−0.41	0.93	0.45
Seizure therapy	−0.56	0.85	−2.23	1.12	0.51
Arthritis deform	0.32	0.58	−0.82	1.46	0.58
Myocardial infarction	0.70	1.32	−1.90	3.31	0.59
Venous thrombosis	0.26	0.50	−0.73	1.24	0.61
Skin extensive scar	−0.43	0.89	−2.18	1.32	0.63
Cardio angina	−0.28	0.72	−1.71	1.15	0.70
Osteomyelitis	0.60	1.98	−3.31	4.52	0.76
Muscle atrophy	−0.22	0.80	−1.80	1.36	0.78
Cognitive impairment	−0.15	0.75	−1.63	1.32	0.84
Pulmonary infarction	0.27	1.98	−3.64	4.19	0.89
Retinal change	−0.04	0.42	−0.88	0.80	0.92

SLICC ACR DI, Systemic Lupus International Collaborating Clinics/American College of Rheumatology Damage Index.

## Discussion

Fatigue is the most common and bothersome symptom reported by patients with SLE.^3 33^ This study examined the association between demographics, disease activity, disease damage accrual and fatigue severity in SLE. The study accounts for multiple factors in a single model to demonstrate the viability of relationships between determinant factors and SLE-related fatigue, to help understand the pathophysiological mechanism of fatigue. The study cohort reported significant fatigue despite low disease activity. There were significant differences in self-reported fatigue among various races; most non-Hispanic White patients (71%) had FSS scores that were≥4; conversely, a much smaller proportion of Black patients (40%) reported clinically significant fatigue. Hispanic and Asian patient groups were equally divided between low vs high fatigue scores. The results from our study suggest that organ damage accrual, specifically manifested as neuropathy and pulmonary fibrosis, and body mass are significantly associated with the severity of fatigue in patients with SLE. Interestingly, SLE disease duration was not associated with SLE-related damage and clinically significant fatigue.

The mechanism underlying fatigue in SLE is not well understood. Recently published data suggest patients can be stratified into those with a high degree of fatigue and low disease activity, high degree of fatigue and high disease activity, and low disease activity and low degree of fatigue, and the use of glucocorticoids, anxiety and depression were independent predictors of fatigue.^34^ This suggests that the pathophysiological mechanism responsible for fatigue may be different in various subsets of SLE patients.

The current study found a significant association between pulmonary fibrosis and fatigue, indicating that fatigue in a subset of SLE patients is due to poor cardiopulmonary function. Results from several studies in SLE patients undergoing moderate aerobic exercise have shown a significant improvement in fatigue, possibly due to an improved cardiorespiratory function.^335^,^37^ Another significant and interesting association was found between neuropathy and SLE-related fatigue. Fatigue has been reported in up to 80% of patients with immune-mediated peripheral neuropathy, and it seems to persist despite improvement of underlying neurological function.^38^ There are several reasons for fatigue in patients with neuropathy, such as muscle weakness. The patients with peripheral neuropathy are shown to have reduced drive from their cortical neurons as well as fewer peripheral nerve axons innervating large numbers of muscle fibres.^39^ As a result, patients with peripheral neuropathy are unable to fully activate muscles during voluntary movements, requiring more effort to perform activities of daily living, resulting in fatigue. Furthermore, other factors such as use of orthoses and walking aids needed to compensate for neuropathy and muscle weakness may also contribute to fatigue. The prevalence of peripheral neuropathy is reported to be present in up to 14% of SLE patients; however, an association with fatigue in SLE patients has not been described previously.^40^

Over a period of time, the underlying cardiorespiratory dysfunction and peripheral neuropathy can create a fear of precipitating fatigue, which can lead to an avoidance of physical activity and physical deconditioning, further exacerbating fatigue.^41^

In agreement with previous reports, higher BMI in the overweight and obesity range was found to be significantly associated with fatigue severity.^27 42 43^ This association needs to be further explored since increased BMI may result from reduced physical activity due to fatigue.^44^ In a recent study, being overweight contributed to adverse physical and mental HRQoL outcomes, including fatigue, despite successful therapeutic intervention to control SLE disease activity.^45^ Other potential causes of increased BMI include poor diet and comorbid conditions, including depression, hypothyroidism, cardiovascular disease and respiratory disease.

Our study found that higher fatigue was more prevalent in non-Hispanic White patients, contrary to data reported in the non-SLE-related fatigue population.^46^ Furthermore, we found that Black patients were less likely to report higher degrees of fatigue as compared with other ethnic groups. Previous studies have reported mixed results, with some suggesting an overall worse HRQoL and fatigue in Black lupus patients and other studies showing higher disease activity, but a lower degree of fatigue reported by Black lupus patients.^47^ The possible explanations for these conflicting results are the use of different tools to measure fatigue, treatment settings, socioeconomic domains and differences in the demographic characteristics of the cohorts. The socioeconomic background information of our patients was not collected during this study. Differences in fatigue among various racial groups need to be confirmed in larger longitudinal studies of multi-ethnic cohorts, looking at the various social, structural, disease-related factors and their impact on fatigue.

In the current study, disease activity as measured by SELENA-SLEDAI was found to be unrelated to fatigue severity. This finding was consistent with previous studies using SELENA-SLEDAI as a measure of disease activity.^548^,^50^ In a longitudinal study of 280 lupus patients, fatigue and disease activity were found to follow distinct trajectories, and disease activity alone could not fully explain fatigue trajectories.^51^ Conversely, using the Systemic Lupus Activity Measure (SLAM) as the disease activity measure, Tench *et al* observed a strong association between fatigue severity and disease activity, even when the fatigue variable was removed from calculated SLAM scores to prevent skewed results.^52^ We did not find any association of fatigue with the serum complement proteins, use of corticosteroid and hydroxychloroquine, which is consistent with some of the published studies.^1 2 49 50^ Conversely, data from the Swiss SLE Cohort Study showed that low complement and/or the presence of anti-dsDNA antibodies were associated with increased fatigue and reduced mental health.^53^

In a previous study by Bruce *et al*, with a smaller sample size, a significant correlation was found with tender point count and quality of life as measured by SF-36, but no correlation was found with SLICC ACR DI damage or SLEDAI scores.^5^ Another study of 102 lupus patients from Spain found a significant association between the presence of fatigue and fibromyalgia, as well as multiple other factors such as age, Multidimensional Health Assessment Questionnaire, SLICC ACR DI, photosensitivity, the summer season and vitamin D insufficiency.^54^ In the current study, we did not classify SLE subjects with fibromyalgia, as the study team considered that SLE patients with fatigue would meet the exclusion criteria based on the 2010 American College of Rheumatology diagnostic criteria for fibromyalgia.^55^

There were several limitations of our study. The data were collected at a single time point in a relatively small sample size, with the majority of patients having low disease activity, limiting generalisation of the findings. We used translators in rare instances when the instrument was not available in the patient’s native language, raising the possibility of inaccurate data. Subjects were not stratified by physical activity levels, pain, insomnia, anxiety, depression and stress levels, intensity and frequency of flares, or other comorbidities that may exacerbate fatigue.^56^ Additionally, some patients were on medications that could have confounded the perceptions of fatigue, such as anti-hypertensive and antidepressant medications. The current study characterised fatigue based on patients’ self-report; therefore, any inference made must be restricted to subjects’ overall perception of fatigue rather than fatigue experienced in response to any particular type of activity (physical, mental or social).

In summary, our study identified that clinically significant fatigue in SLE is associated with organ damage accrual, specifically due to pulmonary fibrosis, neuropathy and high BMI. Black patients as a group reported less fatigue with the same degree of damage accrual. Fatigue was found to be not associated with lupus disease activity or glucocorticoid treatment. We are collecting longitudinal data to confirm findings from this cross-sectional cohort. Mechanistic studies are needed to understand the pathophysiology of fatigue in various subsets of SLE patients.

## Supplementary material

10.1136/bmjopen-2025-104786 online supplemental file 1

## Data Availability

Data are available on reasonable request.
