# Supplementary material for: Factors associated with fatigue in patients with systemic lupus erythematosus in an outpatient tertiary care setting: a cross-sectional cohort study
Source: BMJ Open. 2026 Apr 1;16(4):e104786. doi: 10.1136/bmjopen-2025-104786 (PMC13052649; doi:10.1136/bmjopen-2025-104786)
Supplement: online supplemental file 1 [file bmjopen-16-4-s001.docx]

Supplemental Tables

Supplement Table1. Laboratory results of the participants (N=183)

| Sodium, Mean (SD) mmol/L | 140.5( 2.5) |
| --- | --- |
| Potassium, Mean (SD) mmol/L | 3.9( 0.4) |
| Chloride, Mean (SD) mmol/L | 102.5( 3.0) |
| Creatinine, Mean (SD) mg/dl | 0.9( 1) |
| e Glomerular Filtration Rate (eGFR) African American, Mean (SD) | 101.1( 26.3) |
| e Glomerular Filtration Rate (eGFR) non-African American, Mean (SD) | 91.6( 26.4) |
| Glucose, Mean (SD) mg/dl | 92.6( 30.6) |
| Blood Urea Nitrogen (BUN), Mean (SD) | 15.7( 9.4) |
| Albumin, Mean (SD) g/dl | 4.1( 0.4) |
| Calcium, Mean (SD) mg/dl | 2.3( 0.1) |
| Magnesium, Mean (SD) mg/dl | 0.8( 0.1) |
| Phosphorus, Mean (SD) mg/dl | 3.4( 0.7) |
| Alkaline Phosphatase, Mean (SD) U/L | 73.8( 25.3) |
| Alanine Aminotransferase (ALT), Mean (SD) U/L | 21.4( 15.3) |
| Aspartate Aminotransferase (AST), Mean (SD) U/L | 24.1( 12.2) |
| Bilirubin Total, Mean (SD) mg/dl | 0.4( 0.2) |
| Bilirubin Direct, Mean (SD) mg/dl | 0.2( 0.1) |
| Lactate Dehydrogenase (LDH), Mean (SD) U/L | 198.8( 60.5) |
| Protein Total, Mean (SD) g/dl | 7.4( 0.7) |
| Creatine Kinase, Mean (SD) U/L | 105.9( 116.8) |
| Uric Acid, Mean (SD)mg/dl | 5.3( 1.7) |
| Cholesterol, Mean (SD) mg/dl | 176.3( 41.4) |
| High-Density Lipoprotein (HDL), Mean (SD) mg/dl | 62.6( 22) |
| Low-Density Lipoprotein (LDL), Mean (SD) mg/dl | 90.1( 33.5) |
| Triglycerides, Mean (SD) mg/dl | 118.5( 62.5) |
| C-Reactive Protein, Mean (SD) mg/L | 4( 7.7) |
| White Blood Cell count, Mean (SD) K/mcL | 5.6( 2.1) |
| Red Blood Cell count, Mean (SD) M/mcL | 4.3( 0.6) |
| Hemoglobin, Mean (SD) g/dL | 12.3( 1.5) |
| Hematocrit, Mean (SD) % | 37.7( 4.3) |
| Platelet, Mean (SD) K/mcL | 240.9( 75.0) |

Supplement Table 2. SELENA-SLEDAI domains in subjects with SLEDAI score >4

| SLEDAI Domains | Number of subjects |
| --- | --- |
| Arthritis | 9 |
| Hematuria | 2 |
| Proteinuria | 11 |
| Pyuria | 1 |
| Rash | 13 |
| Alopecia | 12 |
| Mucosal Ulcers | 3 |
| Low Complement | 29 |
| Increased anti-ds DNA Binding | 27 |
| Fever | 1 |
| Thrombocytopenia | 1 |
| Leukopenia | 4 |

Supplement Table 3. Comparison of laboratory data for subjects with low vs high fatigue

|  | FSS Low (FSS<4) | FSS High (FSS≥4) | All Subjects | *p*-value |
| --- | --- | --- | --- | --- |
|  | (N=88) | (N= 95) | (N=183) |  |
| Sodium mmol/L |  |  |  |  |
| Mean (SD) | 140.6( 2.3) | 140.4( 2.6) | 140.5( 2.5) | 0.46 |
| Potassium mmol/L |  |  |  |  |
| Mean (SD) | 4( 0.4) | 3.9( 0.4) | 3.9( 0.4) | 0.28 |
| Chloride mmol/L |  |  |  |  |
| Mean (SD) | 102.6( 2.4) | 102.4( 3.4) | 102.5( 3.0) | 0.57 |
| Creatinine mg/dL |  |  |  |  |
| Mean (SD) | 1( 1.5) | 0.8( 0.3) | 0.9( 1.0) | 0.12 |
| e Glomerular Filtration Rate (eGFR) African American, |  |  |  |  |
| Mean (SD) | 99.1( 29.7) | 103( 22.6) | 101.1( 26.3) | 0.33 |
| e Glomerular Filtration Rate (eGFR), non-African American, |  |  |  |  |
| Mean (SD) | 90.1( 29.5) | 93( 23.2) | 91.6( 26.4) | 0.47 |
| Glucose mg/dL |  |  |  |  |
| Mean (SD) | 89.2( 12.4) | 95.9( 40.8) | 92.6( 30.6) | 0.13 |
| Blood Urea Nitrogen (BUN) mg/dL |  |  |  |  |
| Mean (SD) | 16.5( 11) | 14.9( 7.5) | 15.7( 9.4) | 0.27 |
| Albumin g/dL |  |  |  |  |
| Mean (SD) | 4.1( 0.4) | 4( 0.4) | 4.1( 0.4) | 0.04 |
| Calcium mg/dL |  |  |  |  |
| Mean (SD) | 2.3( 0.1) | 2.3( 0.1) | 2.3( 0.1) | 0.34 |
| Magnesium mg/dL |  |  |  |  |
| Mean (SD) | 0.8( 0.1) | 0.8( 0.1) | 0.8( 0.1) | 0.68 |
| Phosphorus mg/dL |  |  |  |  |
| Mean (SD) | 3.5( 0.8) | 3.3( 0.5) | 3.4( 0.7) | 0.12 |
| Alkaline Phosphatase U/L |  |  |  |  |
| Mean (SD) | 73.5( 24.3) | 74( 26.3) | 73.8( 25.3) | 0.90 |
| Alanine Aminotransferase (ALT) U/L |  |  |  |  |
| Mean (SD) | 21.6( 15.4) | 21.1( 15.3) | 21.4( 15.3) | 0.83 |
| Aspartate Aminotransferase (AST) U/L |  |  |  |  |
| Mean (SD) | 24.1( 11.3) | 24( 13.2) | 24.1( 12.2) | 0.96 |
| Bilirubin Total mg/dL |  |  |  |  |
| Mean (SD) | 0.4( 0.2) | 0.4( 0.2) | 0.4( 0.2) | 0.40 |
| Bilirubin Direct mg/dL |  |  |  |  |
| Mean (SD) | 0.3( 0.1) | 0.2( 0.1) | 0.2( 0.1) | 0.54 |
| Lactate Dehydrogenase (LDH) U/L |  |  |  |  |
| Mean (SD) | 204.6( 73.9) | 194.2( 47.1) | 198.8( 60.5) | 0.42 |
| Protein Total g/dL |  |  |  |  |
| Mean (SD) | 7.4( 0.7) | 7.3( 0.6) | 7.4( 0.7) | 0.27 |
| Creatine Kinase U/L |  |  |  |  |
| Mean (SD) | 127.5( 158.9) | 85.1( 41.6) | 105.9( 116.8) | 0.02 |
| Uric Acid mg/dL |  |  |  |  |
| Mean (SD) | 5.5( 1.7) | 5( 1.7) | 5.3( 1.7) | 0.15 |
| Cholesterol mg/dL |  |  |  |  |
| Mean (SD) | 179.6( 46.4) | 173.2( 36.1) | 176.3( 41.4) | 0.31 |
| High-Density Lipoprotein (HDL) mg/dL |  |  |  |  |
| Mean (SD) | 64( 23.7) | 61.2( 20.3) | 62.6( 22) | 0.41 |
| Low-Density Lipoprotein (LDL) mg/dL |  |  |  |  |
| Mean (SD) | 92.2( 36.3) | 88.2( 30.9) | 90.1( 33.5) | 0.43 |
| Triglycerides mg/dL |  |  |  |  |
| Mean (SD) | 118.5( 64.9) | 118.6( 60.6) | 118.5( 62.5) | 0.99 |
| C-Reactive-Protein mg/L |  |  |  |  |
| Mean (SD) | 3.2( 4.4) | 4.8( 9.7) | 4( 7.7) | 0.15 |
| White Blood Cell count (WBC) K/mcL |  |  |  |  |
| Mean (SD) | 5.6( 1.8) | 5.7( 2.4) | 5.6( 2.1) | 0.75 |
| Red Blood Cell count (RBC) K/mcL |  |  |  |  |
| Mean (SD) | 4.3( 0.6) | 4.2( 0.6) | 4.3( 0.6) | 0.56 |
| Hemoglobin g/dL |  |  |  |  |
| Mean (SD) | 12.3( 1.6) | 12.2( 1.4) | 12.3( 1.5) | 0.77 |
| Hematocrit % |  |  |  |  |
| Mean (SD) | 37.9( 4.4) | 37.6( 4.2) | 37.7( 4.3) | 0.65 |
| Platelet K/mcL |  |  |  |  |
| Mean (SD) | 239.7( 68.5) | 242( 80.9) | 240.9( 75.0) | 0.84 |
