# Supplementary material for: Factors associated with fatigue in patients with systemic lupus erythematosus in an outpatient tertiary care setting: a cross-sectional cohort study
Source: BMJ Open. 2026 Apr 1;16(4):e104786. doi: 10.1136/bmjopen-2025-104786 (PMC13052649; doi:10.1136/bmjopen-2025-104786)
Supplement: Supplementary file 2 [file bmjopen-2025-104786.reviewer_comments.pdf]

PEER REVIEW HISTORY

BMJ Open publishes all reviews undertaken for accepted manuscripts. Reviewers are asked to complete a checklist review form and are provided with free text boxes to elaborate on their assessment. These free text comments are reproduced below.

ARTICLE DETAILS

Title (Provisional)

Factors Associated with Fatigue in Patients with Systemic Lupus Erythematosus in an Outpatient Tertiary Care Setting-A cross-sectional cohort study

Authors

Chapman, Marquis; Rizvi, Ali M; Qutab, Miraa; Munday, Adam; Biehl, Ann; Manna, Zerai; Hasni, Sarfaraz

---

VERSION 1 - REVIEW

**Reviewer**	**1**
**Name**	**Fritsch-Stork, Ruth D. E.**
**Affiliation**	**Univ Med Ctr, Rheumatology**
**Date**	**13-Jul-2025**
**COI**	**None**

The paper „Fatigue in Systemic Lupus Erythematosus Is Associated with Damage Accrual and Body Mass Index" by Chapman et al. examines 183 SLE patients of mostly Hispanic or African American descent from one tertiary center in terms of fatigue and its associated factors. The authors find that the group with a higher FSS scores ≥ 4 (N=95) included a higher proportion of caucasian patients with more organ damage, esp. pulmonary fibrosis and neuropathy) and higher BMI.

The paper is well written, focuses on a very prevalent problem in SLE patients and gives a clear conclusion.

There are some aspects I do miss:

- Principally the patients´ characteristics should be given in more detail: no mention of any immunosuppression other than Glucocorticoids, … what was the percentage of patients in remission? Although the SLEDAI is indeed low for all patients, were there any differences in residual activity? Was there only serological or also clinical activity?

- The association of neuropathy and fatigue is well discussed, however, did the authors go back to their patients and ask the e.g. if they had ortheses? The theoretical reasons listed in the discussion should be applied to the patient group.

- The same holds true for the associated BMI and the possibly underlying factors depression, hypothyroidism, cardiovascular disease, and respiratory disease.

- Again, the socioeconomic background in the different ethnic population is missing and should be also taken into account in the results and mentioned.

Minor point:

- Some spaces are missing, please check the formatting of the manuscript.

**Reviewer**	**2**
**Name**	**Pettersson, Susanne**
**Affiliation**	**Karolinska Universitetssjukhuset, Tema Inflammation och infektion**
**Date**	**08-Sep-2025**
**COI**	**None**

Thank you for giving me the opportunity to review the manuscript bmjopen-2025-104786_"Fatigue in Systemic Lupus Erythematosus Is Associated with Damage Accrual and Body Mass Index"

I have some minor questions and suggestions for improvement of the manuscript.

General/formatting:

According to the formatting instructions of BMJ Open the main text headings and subheadings should follow the hierarchy: BOLD CAPS, bold lower case, Plain text. (see Formatting your paper - BMJ Author Hub) I suggest that you correct your manuscript according to the journals instructions.

Tables: you use both comma (.), line (–) and blank space () when referring to the tables in the main text, but there is also differences in your table headings, please be consistent.

Table 2: It is difficult to understand what comparison the p-value for the portions is corresponding to. Is the p-value a comparison between the levels of fatigue or groups of ethnicity? I suggest a clarification in the table, e.g. use a footnote.

According to the instructions of the BMJ open, cross-sectional should follow the STROBE while writing the manuscript. There are some bullets in the STROBE that are not clear how you addressed, eg. How was missing data handled, in the discussion extern validity. So, please check the STROBE and make sure that every bullet in the checklist is addressed in the paper. Link from the author guidelines at the BMJ Open, to STROBE_checklist_BMJ-Open_cross-sectional-studies.docx

Abstract:

When I am looking at abstracts in the latest number of BMJ Open, it seems like the journal do not use colons in the printed abstract's subheadings e.g. after objectives, please suggest according to the formats of the journal.

The abbreviation IRB is not explained in the abstract, I suggest rephrasing or an explanation of the abbreviation, should be in the abstract.

Introduction:

Page 6, the line 38: you state "Pathophysiological mechanisms underlying fatigue in SLE are not well defined". After such sentence I think it would be most scientifically appropriate to refer to research that have studies pathophysiological mechanisms, but I think this is lacking in the submitted paper. So, my suggestion is to either remove this sentence or add some references that support this statement. After this statement you describe and refer to multiple factors, that is fine, but not what kind of mechanisms that have previously, been explored to explain fatigue. Factors and mechanism are not on the same level of scientific explanations. I think the paper would be more solid if you included some kind of conceptual model, or firm explanations of your rational or hypothesis of what factors influence fatigue. Now, it feels a little bit that you only took data that you had, without any rational.

Method:

Make sure that all variables are described properly in the method section, e.g. there are more information on the laboratory parameters in the tables than in the method. Further, You include prednisone in your exploration, please clarify how, e.g. current dose (mg/day) or only yes/no? Please clarify in the method, and in the demographics since the dose of prednisone have known implications on fatigue.

Statistical Analysis: You used Pearsons's correlation to explore relationships, when using Pearsons the variables should be normally distributed, and I question if the variables you explored were normally distributed. Please insert data in the results that support the selected method of correlation, or change statistical method to a non-parametric test.

Further, there are statistical methods presented in e.g. tables that are not in the method section. Please make sure that all statistical methods used in your study are described in the method section, for transparency.

Page 9, Line 10: You write that the Stepwise multivariable regression analysis was adjusted for key confounders. However, I can not find in the main-text or in the corresponding table what confounders that the model was adjusted for, please clarify. Additionally I think it would be appropriate to include the hypothesis of these confounders.

Results:

The descriptive results of all variables should be presented either in the text of in table 1. E.g. I am missing a description of the laboratory results and medication that are included in

the models. And in order to understand the results I think this is needed, or otherwise please explain why you hide some of your results.

Page 9: You use the wording ”ethnic minorities”, however ethnic minorities are only minorities in countries where other ethnicities are mayor. If no hypothesis related to being of minor ethnical groups, I suggest that you only keep the fact: predominantly Hispanic (43.2%) or African American (AA) (24%) and omit the wordings of minority.

Page 10, line3: You write “The non-Hispanic White subjects reported more fatigue whereas black subjects reported significantly less fatigue”. Less than what? Please complete the sentence. Further you should think of what expressions you use here. As I understand you refer to table 2, the portion within the fatigue groups, not higher median/mean level, thus It would be more appropriate to rephrase the text accordingly.

In the univariate regression model, it is not clear why you only focused on two ethnicities. I suggest all ethnicities should be included in the same way in the model. I found it a little puzzling way the largest ethnicity group is not visual in table 3. Please clarify.

For transparency I suggest that you show also the non-significant data, that can for examples be included as a supplement if you chose to not include this among the results.

Discussion:

Page 11, line 17: You write ” The study uniquely accounts for multiple factors in a single”, I question that it is unique to account for multiple factors while explaining fatigue. Please rephrase and replace the word uniquely. And again you do not explore pathophysiological mechanisms, so I think that you should rewrite the first part of the discussion to more scientifically true.

Page 12, line you state that previous research have note found any associaions between fatigue and complement, please read e.g. Impact of disease activity on health-related quality of life in systemic lupus erythematosus - a cross-sectional analysis of the Swiss Systemic Lupus Erythematosus Cohort Study (SSCS) - PubMed and reconsider your statement.

I miss the discussion or limitation that all participants did not answer he questionnaires themselves. In the method the use of a translator is described. However, we do not know how many times, and to which languages. To use a translator makes the possibility for some patients to be included in the study. To what languages did the translator help the patients? The instruments are validated for several languages so why not use a validated versions. Please include more information and discuss your method and limitations and strengths related to the use of a translator.

VERSION 1 - AUTHOR RESPONSE

1. Reviewer: 1
   Dr. Ruth D. E. Fritsch-Stork, Univ Med Ctr

Comments to the Author:
The paper „Fatigue in Systemic Lupus Erythematosus Is Associated with Damage Accrual and Body Mass Index" by Chapman et al. examines 183 SLE patients of mostly Hispanic or African American descent from one tertiary center in terms of fatigue and its associated factors. The authors find that the group with a higher FSS scores ≥ 4 (N=95) included a higher proportion of caucasian patients with more organ damage, esp. pulmonary fibrosis and neuropathy) and higher BMI.

The paper is well written, focuses on a very prevalent problem in SLE patients and gives a clear conclusion.

There are some aspects I do miss:
- Principally the patients´ characteristics should be given in more detail: no mention of any immunosuppression other than Glucocorticoids, ... what was the percentage of patients in remission?
Response:
We revised Table 1 by adding additional patient characteristics. There were 18 (9.8%) patients on methotrexate, 35(19.1%) on azathioprine, and 47 (25.7%) on mycophenolate mofetil.
A total of 81(44.2%) were considered to be in remission due to SLEDAI ≤ 4 with no clinical disease activity, and Physician's Global Assessment (PGA) of < 0.5.

-Although the SLEDAI is indeed low for all patients, were there any differences in residual activity? Was there only serological or also clinical activity?
Response: There was no difference in SLEDAI scores between patients with FSS scores>4 and <4 (Table 2). There were 35 patients with only serological disease activity, and 46 patients had a SELENA-SLEDAI score =0. We have added a supplemental table listing the positive clinical domains for patients with a SELENA-SLEDAI >4. The majority of the clinical activity was due to rash, alopecia, and renal involvement.

- The association of neuropathy and fatigue is well discussed, however, did the authors go back to their patients and ask the e.g. if they had ortheses? The theoretical reasons listed in the discussion should be applied to the patient group.
Response: Thank you for this insightful comment. We agree that the association between fatigue and neuropathy in SLE should be further explored. Reaching back to our patients about their use of orthoses is logistically not feasible as some of the patients have either relocated or their current contact information is not available.

- The same holds true for the associated BMI and the possibly underlying factors depression, hypothyroidism, cardiovascular disease, and respiratory disease.
Response: We have updated Table 2 to list co-morbid conditions that may contribute to fatigue. Numerically, there were more patients with co-morbid conditions reporting

higher fatigue, but the results were not statistically significant. We have added the following statement reflecting this under the results section:

The majority of patients did not have any co-morbid conditions that could contribute to fatigue. In the subset of patients with depression, hypothyroidism, cardiovascular, or respiratory diseases, there were more who reported clinically significant fatigue, but the results were not statistically significant (Table 2).

- Again, the socioeconomic background in the different ethnic population is missing and should be also taken into account in the results and mentioned.

Response: Thank you for this valuable comment. We agree that socioeconomic background can influence some of the results mentioned. This data is not available for our cohort. We have added this as a limitation of our study. The following statements were added under the results and discussion sections:

- *The information about socio-economic background of our patients was not collected.*
- *The socio-economic background information of our patients was not collected during this study.*

Minor point:

- Some spaces are missing, please check the formatting of the manuscript.

Response: Manuscript checked, and formatting issues were fixed.

2. Reviewer: 2
   Dr. Susanne Pettersson, Karolinska Universitetssjukhuset
   Comments to the Author:
   Thank you for giving me the opportunity to review the manuscript bmjopen-2025-104786_"Fatigue in Systemic Lupus Erythematosus Is Associated with Damage Accrual and Body Mass Index"

   I have some minor questions and suggestions for improvement of the manuscript.
   General/formatting:
   According to the formatting instructions of BMJ Open the main text headings and subheadings should follow the hierarchy: BOLD CAPS, bold lower case, Plain text. (see Formatting your paper - BMJ Author Hub) I suggest that you correct your manuscript according to the journals instructions.

Response: Manuscript formatting done as per instructions on BMJ Author Hub.

Tables: you use both comma (.), line (–) and blank space () when referring to the tables in the main text, but there is also differences in your table headings, please be consistent.
Response: Tables were formatted to be consistent throughout the manuscript and table headings. In addition, tables are placed in the main text where the table is first cited as per the BMJ Author Hub.

Table 2: It is difficult to understand what comparison the p-value for the portions is corresponding to. Is the p-value a comparison between the levels of fatigue or groups of ethnicity? I suggest a clarification in the table, e.g. use a footnote.
Response: The p-value refers to comparison between the levels of fatigue. A clarification footnote was added to Table 2.

According to the instructions of the BMJ open, cross-sectional should follow the STROBE while writing the manuscript. There are some bullets in the STROBE that are not clear how you addressed, eg. How was missing data handled, in the discussion extern validity. So, please check the STROBE and make sure that every bullet in the checklist is addressed in the paper. Link from the author guidelines at the BMJ Open, to STROBE_checklist_BMJ-Open_cross-sectional-studies.docx
Response: A strobe checklist is completed and uploaded with the revised manuscript. There was no missing data for this study due to our comprehensive research data capture and storage system.

Abstract:
When I am looking at abstracts in the latest number of BMJ Open, it seems like the journal do not use colons in the printed abstract's subheadings e.g. after objectives, please suggest according to the formats of the journal.
Response: Abstract reformatted conforming to the BMJ open format by removing colons after subheadings.

The abbreviation IRB is not explained in the abstract, I suggest rephrasing or an explanation of the abbreviation, should be in the abstract.
Response: Explanation of IRB abbreviation added in the abstract.

Introduction:
Page 6, the line 38: you state "Pathophysiological mechanisms underlying fatigue in SLE are not well defined". After such sentence I think it would be most scientifically appropriate to refer to research that have studies pathophysiological mechanisms,

but I think this is lacking in the submitted paper. So, my suggestion is to either remove this sentence or add some references that support this statement. After this statement you describe and refer to multiple factors, that is fine, but not what kind of mechanisms that have previously, been explored to explain fatigue. Factors and mechanism are not on the same level of scientific explanations. I think the paper would be more solid if you included some kind of conceptual model, or firm explanations of your rational or hypothesis of what factors influence fatigue. Now, it feels a little bit that you only took data that you had, without any rational.

Response: Thank you for a very helpful comment. One of the putative mechanisms underlying fatigue is mitochondrial dysfunction in SLE. We are currently conducting a clinical trial to boost NAD+ levels in SLE patients. We have added following mechanisms that have previously been explored to explain fatigue:

Dysregulation of mitochondrial structure and function leads to increased oxidative stress, which contributes to inflammation and fatigue seen in SLE(18). An altered kynurenine pathway, leading to an increased kynurenine/tryptophan ratio, was shown to correlate with fatigue in SLE (19). High levels of quinolinic acid, a metabolite in the kynurenine pathway, were reported in patients with SLE. Quinolinic acid has been shown to initiate inflammation and can generate reactive oxygen species, resulting in fatigue. Quinolinic acid is required for the synthesis of nicotinamide adenine dinucleotide (NAD+), a coenzyme that catalyzes redox reactions in the body (20). Boosting NAD+ blunts attenuated type I IFN signaling in myeloid cells and may improve mitochondrial dysfunction and fatigue in SLE patients(21). A clinical trial to evaluate the effect of NAD+ boosting in SLE is currently underway (NCT06032923).

Method:

Make sure that all variables are described properly in the method section, e.g. there are more information on the laboratory parameters in the tables than in the method. Further, You include prednisone in your exploration, please clarify how, e.g. current dose (mg/day) or only yes/no? Please clarify in the method, and in the demographics since the dose of prednisone have known implications on fatigue.

Response: Thank you for your suggestion. We have added the following under the methods and results section, respectively:

Current prednisone use and dose were recorded on the day of the visit. Lab parameters, including hemoglobin level and complement proteins, with potential effects on fatigue, were obtained on the day of the visit.

The current use and dose of prednisone were similar in both groups.

Statistical Analysis: You used Pearson's correlation to explore relationships, when using Pearson's the variables should be normally distributed, and I question if the variables you explored were normally distributed. Please insert data in the results that support the selected method of correlation, or change statistical method to a non-parametric test.

Further, there are statistical methods presented in e.g. tables that are not in the method section. Please make sure that all statistical methods used in your study are described in the method section, for transparency.
Page 9, Line 10: You write that the Stepwise multivariable regression analysis was adjusted for key confounders. However, I can not find in the main-text or in the corresponding table what confounders that the model was adjusted for, please clarify. Additionally I think it would be appropriate to include the hypothesis of these confounders.

Response: Thank you for your useful comments. We reanalyzed the data using Spearman's rank correlation and the respective tables were updated accordingly. There were no significant changes in the results. The missing methods were added to the statistical analysis section. In addition, information about confounders and the hypothesis for them were added. Below is the revised statistical analysis section:
Subject demographics were characterized and reported by mean, standard deviation, and range for the continuous variables, and frequencies and percentages for the categorical variables. Independent samples t-tests were performed to compare continuous variables between the high and low FSS score groups, while chi-square analyses were conducted to assess differences in categorical variables between the groups. Logistic regression analysis was performed to identify independent predictors of high versus low FSS scores. A multiple regression model was fitted, paying special attention to multicollinearity. Spearman's rank correlation coefficient was used to examine the strength and direction of associations between fatigue and individual organ system scores of the SLICC/ACR Damage Index, as the data were not normally distributed. Stepwise multivariable regression analyses were performed to identify factors associated with fatigue in SLE patients. Individual items from the SLICC/ACR Damage Index were included as candidate predictors, and models were adjusted for key confounders, including age and disease duration, due to their known associations with accumulated organ damage. The independent factors are the continuous variables including SELENA-SLEDAI, SLICC ACR DI, disease duration, BMI and hemoglobin level. Pearson correlation coefficient was used to describe the linear relationship between the drugs, prednisone and hydroxychloroquine, versus the SELENA-SLEDAI, SLICC ACR DI and FSS scores. Results were considered as statistically significant at p < 0.05. All data analyses were carried out with the SAS program, release 9.4 (SAS Institute, Cary, NC, USA).

Results:
The descriptive results of all variables should be presented either in the text of in table 1. E.g. I am missing a description of the laboratory results and medication that are included in the models. And in order to understand the results I think this is needed, or otherwise please explain why you hide some of your results.

Response: We have revised Tables 1 and 2 to include lab results, medications, and co-morbid conditions.

Page 9: You use the wording ”ethnic minorities”, however ethnic minorities are only minorities in countries where other ethnicities are mayor. If no hypothesis related to being of minor ethnical groups, I suggest that you only keep the fact: predominantly Hispanic (43.2%) or African American (AA) (24%) and omit the wordings of minority.

Response: Thank you for your comment. The wording about ethnic minorities was deleted from results.

Page 10, line3: You write “The non-Hispanic White subjects reported more fatigue whereas black subjects reported significantly less fatigue”. Less than what? Please complete the sentence. Further you should think of what expressions you use here. As I understand you refer to table 2, the portion within the fatigue groups, not higher median/mean level, thus It would be more appropriate to rephrase the text accordingly.

Response: Thank you for your comment. The statement was revised as follows for clarity:

The proportion of non-Hispanic White subjects reporting clinically significant fatigue (FSS≥4) was higher as compared to the proportion of black subjects who reported FSS≥4.

In the univariate regression model, it is not clear why you only focused on two ethnicities. I suggest all ethnicities should be included in the same way in the model. I found it a little puzzling way the largest ethnicity group is not visual in table 3. Please clarify.

Response: Thank you for your comment. We conducted a logistic regression analysis to examine factors associated with fatigue in patients with SLE, using high versus low FSS scores as the binary dependent variable. As part of the model evaluation, we first assessed the collective contribution of all ethnicity variables in a single model (Latino vs. Non-Latino, Black vs. Non-Black, White vs. Non-White, and Asian vs. Non-Asian). When entered simultaneously, none of the ethnicity variables reached statistical significance.

Upon closer examination, the white vs. Non-White comparison appeared borderline significant. To further investigate, each ethnicity variable was then evaluated individually. Two comparisons remained non-significant; however, the White vs. Non-White and Black vs. Non-Black variables achieved statistical significance (p = 0.006* and p = 0.03*, respectively). Based on these findings, both the White vs. Non-White and Black vs. Non-Black variables were retained in the final model.

We added following in results section to clarify: *When all ethnicity variables were assessed simultaneously in logistic regression analysis none of them reached statistical significance. On comparing each ethnicity individually, the White vs. Non-White and Black vs. Non-Black variables achieved statistical significance (p = 0.006* and p = 0.03*, respectively). Based on these findings, both the White vs. Non-White and Black vs. Non-Black variables were retained in the final model.*

For transparency I suggest that you show also the non-significant data, that can for examples be included as a supplement if you chose to not include this among the results.
Response: We have revised and updated the tables to show all data in the main manuscript and additional data as supplement.

Discussion:
Page 11, line 17: You write " The study uniquely accounts for multiple factors in a single", I question that it is unique to account for multiple factors while explaining fatigue. Please rephrase and replace the word uniquely. And again you do not explore pathophysiological mechanisms, so I think that you should rewrite the first part of the discussion to more scientifically true.
Response: We revised the statement as follows to be scientifically true:
The study accounts for multiple factors in a single model to demonstrate the viability of relationships between determinant factors and SLE related fatigue, to help understand the pathophysiological mechanism of fatigue.

Page 12, line you state that previous research have note found any associations between fatigue and complement, please read e.g. Impact of disease activity on health-related quality of life in systemic lupus erythematosus - a cross-sectional analysis of the Swiss Systemic Lupus Erythematosus Cohort Study (SSCS) - PubMed and reconsider your statement.
Response: Thank you for bringing this interesting study to our attention. We have revised the discussion section as follows:
We did not find any association of fatigue with the serum complement proteins, use of corticosteroid and hydroxychloroquine which is consistent with some of the published studies (1, 2, 49, 50). Conversely, data from the Swiss SLE Cohort Study showed low complement and/or presence of anti-dsDNA antibodies were associated with increased fatigue and reduced mental health (53).

I miss the discussion or limitation that all participants did not answer he questionnaires themselves. In the method the use of a translator is described. However, we do not know how many times, and to which languages. To use a translator makes the possibility for some patients to be included in the study. To what languages did the translator help the patients? The instruments are validated for several languages so why not use a validated versions. Please include more information and discuss your method and limitations and strengths related to the use of a translator.
Response: Majority of our subjects were either English or Spanish speaking where we

used a validated version of the instruments. In rare instances such as one subject speaking Vietnamese and another speaking Farsi when translator was used. The NIH Clinical Center provides translator services in all languages which prevents us from excluding any subject based on their preferred language. We added following under Methods:
An interpreter was used for non-English speaking subjects when a validated version of the FSS was not available in the preferred language. No subject was excluded from the study based on their language preference.
We added following under limitations in Discussion Section:
We used translators in rare instances when the instrument is not available in the patient's native language, raising the possibility of inaccurate data.
